# Eosinophilic Mucin Rhinosinusitis in Iranian Patients Undergoing Endoscopic Sinus Surgery

**Published:** 2018-11

**Authors:** Jahangir Ghorbani, Ali Hosseini Vajari, Guitti Pourdowlat, Parisa Ghasemi, Yousef Eskandari, Keyvan Ghasemi

**Affiliations:** 1 *Chronic Respiratory Diseases Research Center,National Research Institute of Tubeclosis and Lung Disease (NRITLD), Shahid Beheshti University of Medical Sciences, Tehran, Iran.*; 2 *Department of Otolaryngology,DR. Masih Daneshvari Hospital, Shahid Beheshti University of Medical Sciences, Tehran, Iran.*; 3 *Chronic Respiratory Diseases Research Center, Shahid Beheshti University of Medical Sciences, Tehran, Iran.*; 4 *Medical student ,Student Research Committee,Arak University of Medical Sciences, Arak ,Iran.*

**Keywords:** Chronic, Eosinophilic, Rhinitis, IgE, Sinusitis, Mucin, Nasal polyps, Osteitis

## Abstract

**Introduction:**

Eosinophilic mucin rhinosinusitis is a type of chronic rhinosinusitis (CRS). Diagnosis and treatment of this condition play a significant role in reducing the patients’ clinical symptoms. This type of rhinosinusitis has a higher relapse rate, compared to the other types. This disease is more resistant to treatment and more dependent on corticosteroid therapy, compared to the other types of rhinosinusitis. Regarding this, the present study was designed to evaluate the frequency of eosinophilic mucin rhinosinusitis in patients undergoing sinus surgery in a tertiary referral center and examine some clinical and laboratory characteristics regarding this type of rhinosinusitis.

**Materials and Methods:**

This cross-sectional observational study was performed on patients over the age of 16 years, who were diagnosed with CRS in the otolaryngology clinic of a referral tertiary-level hospital, and were candidates for endoscopic sinus surgery. Based on the detection of eosinophilic mucin, the subjects were divided into two groups of eosinophilic mucin and non-eosinophilic mucin rhinosinusitis (controls). The groups were compared in terms of sino-nasal outcome test (SNOT-22) scores, Lund-Mackay staging scores, osteitis status, immunoglobulin E (IgE) level, and eosinophilia.

**Results:**

In this study, 46 subjects participated, 29 (63%) cases of whom had eosinophilic mucin. The SNOT-22 score and serum IgE level were significantly higher in the eosinophilic mucin group, compared to those in the control group. Osteitis and Lund-Mackay scores were also higher in the eosinophilic mucin group than those in the control group; however, this difference was not statistically significant.

**Conclusion:**

Patients with eosinophilic mucin rhinosinusitis showed a more severe clinical involvement. Seemingly, the Iranian patients have a lower and higher frequency of eosinophilic mucin rhinosinusitis, compared to the patients from the Western countries and East Asia, respectively.

## Introduction

Chronic rhinosinusitis (CRS) is defined as the inflammation of nose and paranasal sinuses for at least 12 weeks. It is one of the most commonly reported diseases among adults with a prevalence of 15%. This condition can significantly reduce the patients' quality of life (1). The CRS consists of a collection of heterogeneous diseases and has two classifications, namely phenotypes and endotypes. Subtypes of the disease functionally and pathologically are different with one another depending on the involvement of a specific molecule or cell (2). 

The CRS phenotypes are defined based on an observable characteristic or trait, such as the presence or absence of polyps. On the other hand, CRS endotypes are defined based on distinct pathophysiologic mechanisms that might be identified by especial biomarkers (3). Subtypes of this disease differ in response to medical interventions. Two major CRS phenotypes are chronic rhinosinusitis with nasal polyps (CRSwNP) and chronic rhinosinusitis without nasal polyps (CRSsNP). Other subtypes are allergic fungal sinusitis, CRS associated with aspirin-exacerbated respiratory disease, and CRS associated with cystic fibrosis (2).

Since the prevalence of certain disorders is higher in patients with CRS, compared to that in normal population, treatment can be more difficult in these patients, leading to an increase in the relapse rate (4). Altered eosinophils function and IgE-mediated processes have been implicated in CRS pathogenesis (5). Eosinophils release major basic protein (MBP), a cytotoxic agent for epithelium in the sinus mucus, but not in the tissue (6). Therefore, eosinophils are important both in tissue and sinus lumen. A subtype of CRS, is CRS with eosinophilic mucin which can be associated with fungal infections in some cases (7-10). Despite extensive studies, fungi have not been found in all the patients with CRS.

Diagnosis and treatment of this type of rhinosinusitis can play a significant role in reducing the patients’ clinical symptoms and adverse consequences of the disease (11). In these patients, relapse is common and resistance to the treatment is frequent. Moreover, these patients are more dependent on corticosteroid therapy, compared to cases with other types of CRS. Patients with eosinophilic mucin rhinosinusitis typically have thick and highly viscous discharges with a light tan to brown or dark green color (12). Histologically, inflammatory cells are mainly eosinophilic and contain Charcot-Leyden crystals (12,13). 

There are limited studies evaluating this variant of CRS in the Iranian population. Regarding this, the present study aimed to examine the frequency and clinical features of eosinophilic mucin rhinosinusitis in patients referring to our otolaryngology clinic. 

## Materials and Methods

This observational cross-sectional study was conducted on 46 Iranian patients over the age of 16 years, referring to the otolaryngology clinic of a referral tertiary center in Tehran, Iran, in a one-year period from August 2016 to August 2017. The study population was selected through a convenience sampling technique. The diagnosis of CRS was based on the guidelines of the American Academy of Otolaryngology (14) and presence of computed tomography (CT) scan findings in favor of mucosal involvement. The patients underwent endoscopic sinus surgery after the unsuccessful pharmacological treatment for at least 4 weeks with oral antibiotics, intranasal corticosteroid spray, and saline irrigation. According to the observation of eosinophilic mucin, the subjects were divided into two groups of eosinophilic mucin and non-eosinophilic mucin rhinosinusitis (controls). The exclusion criteria were: 1) patient’s unwillingness to participate in the study, 2) acute respiratory infection during the previous month, 3) nasal or sinus tumor, 4) antrochoanal polyp, 5) systemic steroid use during the last month, 6) immunodeficiency, 7) cystic fibrosis, 8) mucociliary system malfunction, and 9) HIV infection. 

The Persian version of sino-nasal outcome test (SNOT-22) was completed one day before the surgery (15). The progression of the disease to the surrounding soft tissues and osteitis status (using the global osteitis scoring scale) was determined based on the patient’s CT scan, which was acquired within the last 2 months, and Lund-Mackay score (16). Moreover, the findings of intranasal endoscopic examination were recorded, and the patients were subjected to spirometry. The pulmonary status was determined with respect to asthma, and its intensity was measured by a pulmonologist. Peripheral blood eosinophils and serum immunoglobulin E (IgE) levels were measured. 

The operation was performed based on the principles of endoscopic sinus surgery. During the operation, thick discharges were collected with a curette, while thin discharges were collected with a suction tip and a syringe. The samples were immediately transferred to the laboratory to examine the presence of fungi and eosinophilic mucin. Furthermore, a pathological examination of sinus mucosa or polyp was conducted.

*Statistical analysis*

Statistical analyses were performed in SPSS software, version 18. The quantitative variables were presented as mean and standard deviation, while the qualitative variables were expressed as absolute and relative frequencies. The data were analyzed using the Kolmogorov-Smirnov test, Independent sample t-test, Chi-square test, and Fisher’s exact test. P-value less than 0.05 was considered statistically significant.

## Results

In the present study, 46 subjects were recruited with the age range of 16-69 years. The patients had the mean age of 40±12 years in eosinophilic mucin and 42±11 in non- eosinophilic mucin . Overall, 60% of the cases were male. All the studied patients were diagnosed with polyps on the endoscopic examinations (Table.1). 

**Table 1:** Frequency of eosinophilic mucin according to different demographic, clinical, and para-clinical characteristics of patients

**Patient’s characteristics**		**Presence of eosinophilic mucin**			
		**No**		**Yes**	
		**Count**	**Percent %**	**Count**	**Percent %**
Gender	Male	10	35.7%	18	64.3%
	Female	7	38.9%	11	61.1%
Asthma	Negative	8	34.8%	15	65.2%
	Mild	5	41.7%	7	58.3%
	Moderate	3	30.0%	7	70.0%
	Severe	1	100.0%	0	0.0%
Soft tissue involvement	No	17	37.0%	29	63.0%
	Yes	0	0.0%	0	0.0%
Immunoglobulin E	Normal	13	52.0%	12	48.0%
	High	4	19.0%	17	81.0%
Serum Eosinophil	Normal	4	66.7%	2	33.3%
	High	13	32.5%	27	67.5%
Fungal smear	Negative	17	40.5%	25	59.5%
	Positive	0	0.0%	4	100.0%
Osteitis	Negative	3	42.9%	4	57.1%
	Mild	8	47.1%	9	52.9%
	Moderate	4	26.7%	11	73.3%
	Severe	2	28.6%	5	71.4%
					

Eosinophilic mucin was detected in 63% of the patients on the pathological examinations. On the other hand, 37% of the participants were diagnosed with non-eosinophilic mucin chronic rhinosinusitis (Table.2).

**Table 2:** Quantitative variables in patients with and without eosinophilic mucin

**Patient’s Characteristics**	**Presence of eosinophilic mucin**								
	**No**				**Yes**				
	**Mean**	**Standard Deviation**	**Minimum**	**Maximum**	**Mean**	**Standard deviation**	**Minimum**	**Maximum**	**P-value**
Age	42	11	32	69	40	12	16	69	0.634
Lund-McKay Score	20	4	12	24	20	5	6	24	0.956
Eosinophilia	7	13	0	50	43	19	10	80	0.001*
Sino-nasal outcome test-22	38	12	26	68	54	20	25	90	0.004*

* Significant at <0.005

**Fig 1:**
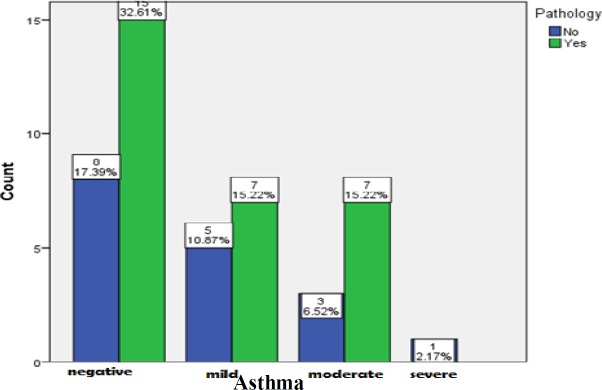
Frequency of eosinophilic mucin in patients with different stages of asthma

According to statistical analysis, the age difference was not significant between the groups (P=0.672). Based on the results of the Chi-square test, the difference between the groups was not significant in terms of gender (P>0.999). As the findings indicated, 50% of the participants suffered from asthma. However, based on the Fisher’s exact test, the difference between the groups was not significant regarding asthma (P=0.613) (Fig.1).

**Fig2:**
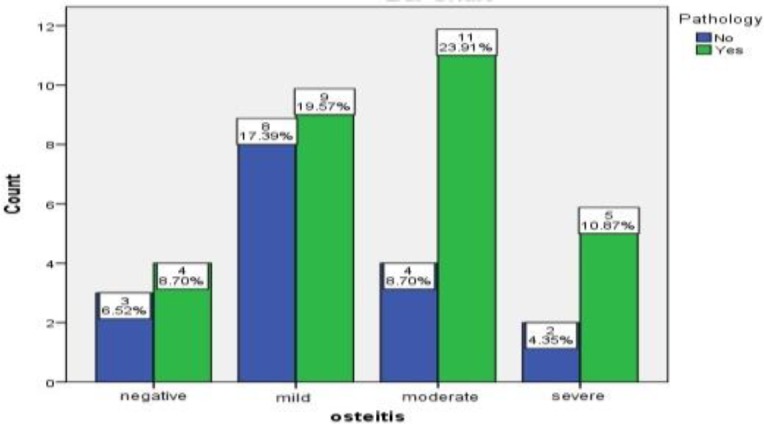
Frequency of eosinophilic mucin in patients with different stages of osteitis

In the examination of osteitis status, 84% of the subjects were diagnosed with osteitis, while 15% of cases were not detected with osteitis. Among the patients with osteitis, 16%, 32%, and 15% of the cases were mild, moderate, and severe, respectively. The intensity of osteitis was defined according to the global osteitis scoring scale based on the patients’ CT scans. The difference between the two pathological groups regarding osteitis status was not significant (P=0.705). Statistically, 64% of the patients with eosinophilic mucin had osteitis[with variant score included mild, moderate and severe], while only 35.8% of the cases with non-eosinophilic mucin had osteitis with different scores (Fig. 2).

**Fig 3:**
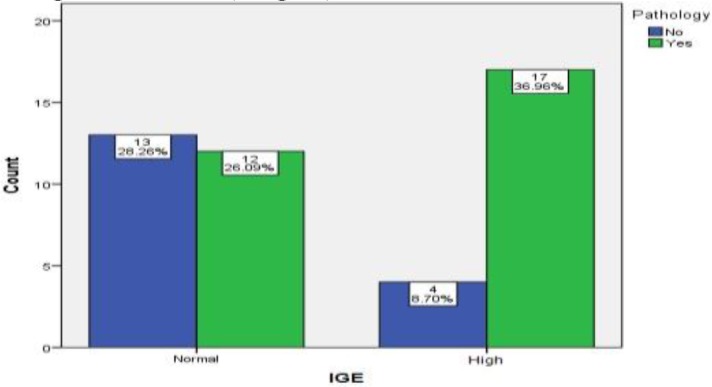
The frequency of eosinophilic mucin in patients with normal and high serum immunoglobulin E

The mean score of SNOT-22 was 48.11 among the participants. There was a significant association between SNOT-22 score and eosinophilic mucin. In this regard, the patients with eosinophilic mucin had higher SNOT-22 scores (P=0.004), which indicated more severe clinical symptoms in these cases. Moreover, the mean Lund-Mackay score was 19.7, which was not significantly [difference between the two groups] (P=0.956) based on the t-test results. However, this score was slightly higher in patients with eosinophilic mucin than in controls (Table.2).The mean eosinophil count in the peripheral blood was obtained as 29.4. Regarding the eosinophil count, the difference was not significant between the eosinophilic mucin and non-eosinophilic mucin groups (P=0.174). In this study, 54% of the patients had normal IgE levels, while 46% of them had high IgE levels (Fig.3).

The IgE level was significantly different between these two pathological groups, and participants with eosinophilic mucin had higher IgE levels (P=0.001). Regarding the clinical and imaging findings, none of the participants showed the involvement of adjacent soft tissues. Furthermore, in the eosinophilic mucin group, only four cases (7%) were positive for fungus on smear and culture.

## Discussion

Diagnosis of the patients with eosinophilic mucin rhinosinusitis can change the treatment plans, since this type of the rhinosinusitis is more resistant to treatment (12). This CRS variant is considered a systemic disease, and systemic corticosteroid therapy is a part of treatment for this condition. The eosinophils in the mucus originate from the tissues. When a large number of these cells are present in the tissue, they can be abundantly found in the mucus, especially as clusters (17).

 In a study performed by Uri et al., 34 patients were examined, 26 cases of whom had eosinophilic mucin rhinosinusitis, while eight cases had allergic fungal rhinosinusitis. In the mentioned study, orbital involvement was greater in the group with allergic fungal rhinosinusitis (50% vs. 7.7%). Nonetheless, the frequency of asthma was reported to be lower in participants with allergic fungal rhinosinusitis (37% vs. 73%) (13). In the present study, the difference between the groups was not significant regarding asthma. Similar to the results reported by Uri et al., the adjacent tissue involvement was not significantly different between the two groups.

Ferguson divides eosinophilic mucin rhinosinusitis into two groups of eosinophilic mucin rhinosinusitis and allergic fungal rhinosinositis . These groups are histologically similar, while fungal elements are not observed in the former group (18). Furthermore, Ponikau et al., using novel and precise techniques of fungus culture from nasal secretions, reported a fungal prevalence of 96%, which is different from the prevalence rate reported in our study (7%). In the mentioned study, the prevalence of the allergic mucin was estimated as 96% (19).

Moreover, in a study conducted by Lara and Gomez, chronic rhinosinusitis with eosinophilic mucus was reported in 25 out of 400 (6.25%) patients who underwent the surgery. However, this prevalence is lower than those in other reports from the North America (10). Some of these differences can be explained by variations in fungal and eosinophil examination methods, multiple definitions of eosinophilia, and difficulty of collecting nasal samples. Furthermore, in a study carried out by Braun et al. in Europe, the prevalence rates of fungal species and eosinophilic mucin were reported as 91.3% and 94.6%, respectively. These rates are significantly different from those obtained in the present study (17).

In a study performed in China, the prevalence of non-eosinophilic rhinosinusitis in patients with chronic rhinosinusitis with polyp was more than 50% (20). in In another study, eosinophilic infiltration polyps was less severe in the Chinese patients, compared to those in the Caucasian patients(21). In addition, in a Thai study, out of 214 patients requiring sinus surgery, 6 cases were reported to have eosinophilic mucin rhinosinusitis (22). In a study conducted in Japan, 88 out of 130 participants had non-eosinophilic mucin. In the mentioned study, the eosinophil count in the blood, IgE level, clinical symptoms, and severity of imaging results were higher in the eosinophilic mucin group (23). According to the results of the mentioned studies, eosinophilic infiltration in polyps of non-Caucasian patients was less significant (23). 

The present research is the first attempt to analyze eosinophilic mucin in the Iranian patients with CRS and polyp. The frequency of eosinophilic mucin rhinosinusitis in our participants was lower than the rates reported in North American and European studies; nonetheless, the rates were significant. In terms of the frequency, the Iranian patients stand between the Caucasian and Oriental cases. In the present study, more than half of the patients had eosinophilic mucin with higher SNOT-22 scores; therefore, compared to other patients with rhinosinusitis, they showed more severe symptoms. 

The main strength of this study is the use of such instruments as the global osteitis scoring scale and SNOT-22, which have not been applied in many previous studies. Particularly, high SNOT-22 scores in patients with eosinophilic mucin objectively indicated the poor clinical presentations of these patients. These clinical differences between the groups can suggest the classification of CRS with polyp into two groups of eosinophilic and non-eosinophilic; in addition, this suggestion has been disclosed in other studies (24). 

Polyposis patients with thick discharge, high peripheral blood eosinophil count, and especially high serum IgE levels (20) have a risk of eosinophilic chronic rhinosinusitis. The cases should be informed before the surgery about the prognosis, and they need to simultaneously apply the medical and surgical treatments and consider the possibility of relapse. According to Sakuma et al., in addition to high eosinophil count, other predictors of eosinophilic mucin rhinosinusitis include the involvement of posterior ethmoid cells and olfactory cleft (24). With respect to treatment, the use of monoclonal antibodies against eosinophilic antigens may be a proper option for the treatment of this disease (25).

One of the limitations of the present study is the consideration of only one tertiary-level referral center. Therefore, it is recommended to conduct further studies on several pathological groups at different centers using a larger sample size and more accurate diagnostic methods (especially for fungal prevalence) in order to analyze eosinophilic mucin rhinosinusitis more precisely. Another limitation of this study is the diversity of pathological definitions, as well as the wide variety of eosinophil and fungal assessment methods in various studies, which can make the comparison difficult. 

## Conclusion

This study revealed a significantly direct correlation between eosinophilic mucin and high SNOT-22 score and serum IgE level in CRS patients. This group of patients also had higher osteitis frequency and Lund-Mackay scores; nonetheless, based on the statistical tests, the differences were not statistically significant. More than half of the cases with CRS and polyp, who referred to our clinic, had eosinophilic mucin. 
